# Intraductal Papillary Neoplasms of the Bile Duct: Clinical Case Insights and Literature Review

**DOI:** 10.3390/clinpract14050133

**Published:** 2024-08-27

**Authors:** Luca Toti, Tommaso Maria Manzia, Francesca Di Giuliano, Eliseo Picchi, Laura Tariciotti, Domiziana Pedini, Luca Savino, Giuseppe Tisone, Roberta Angelico

**Affiliations:** 1 HPB and Transplant Unit, Department of Surgical Sciences, University of Rome Tor Vergata, 00133 Rome, Italy; manzia@med.uniroma2.it (T.M.M.); domiziana.pedini@ptvonline.it (D.P.); tisone@med.uniroma2.it (G.T.); roberta.angelico@gmail.com (R.A.); 2 Diagnostic Imaging Unit, Department of Biomedicine and Prevention, University of Rome Tor Vergata, 00133 Rome, Italy; francesca.di.giuliano@uniroma2.it (F.D.G.); eliseo.picchi@uniroma2.eu (E.P.); 3 UOC HPB and Transplant Unit, Policlinico Tor Vergata, 00133 Rome, Italy; laura.tariciotti@ptvonline.it; 4 Institute of Anatomic Pathology, Department of Biomedicine and Prevention, University of Rome Tor Vergata, 00133 Rome, Italy

**Keywords:** intraductal papillary neoplasm of the bile duct, cholangiocarcinoma, liver resection

## Abstract

Background: Intraductal papillary neoplasms of the bile duct (IPNB) are rare precancerous lesions with implications for the development of cholangiocarcinoma (CCA). Recognizing IPNB and managing its recurrence pose challenges in clinical practice. We present two cases. Case 1: a 60-year-old man presented with an 8 × 8 × 9 cm hepatic cyst initially suspected to be a hydatid cyst. Histology post-resection revealed an IPNB with foci of adenocarcinoma. Despite negative oncologic margins, recurrence occurred eight years later as an rT2N0 lesion. Surgical resection was performed without adjuvant chemotherapy, resulting in the patient’s survival at 48 months post-surgery. Case 2: a 28-year-old female with cognitive impairment was admitted with pulmonary embolism and a liver lesion diagnosed as a simple cyst. Subsequent evaluation revealed adenocarcinoma with local metastases, extensive vascular involvement, and thrombosis. Despite aggressive management, including thrombectomy and chemotherapy, the patient’s condition deteriorated, leading to hepatic failure and eventual demise. Conclusion: IPNB represents a rare premalignant subtype with a propensity for progression to CCA. R0 surgical resection typically offers favorable oncological outcomes with a minimal recurrence risk. Surgical intervention for localized resectable recurrence is both safe and feasible. International registries tracking IPNB recurrence are essential for advancing understanding and optimizing diagnosis, management, and treatment strategies.

## 1. Introduction

Intraductal papillary neoplasms of the bile duct (IPNB) are rare premalignant lesions that affect the biliary ducts and have distinct clinicopathological features [1,2]. Unlike intraductal papillary neoplasms of the pancreas (IPMN), IPNBs have a higher histological grade and advanced stage, which increases the risk of progression to invasive cholangiocarcinoma (CCA) [3].

Recognized as an independent entity by the WHO, the incidence of IPNBs varies geographically, with improved diagnostic methods facilitating their identification [4].

The development of IPNBs is linked to factors such as primary sclerosing cholangitis (PSC), choledochal cysts, and hepatolithiasis. It likely involves a combination of genetic, environmental, and inflammatory factors, including chronic biliary inflammation and genetic alterations [5].

Histologically, IPNBs show intraductal papillary growth with varying degrees of dysplasia, from low-grade to invasive carcinoma. They are subclassified based on histological features, such as pancreatobiliary, intestinal, gastric, and oncocytic subtypes, and further characterized by mucin production [6].

Clinically, IPNBs present nonspecific symptoms, ranging from abdominal pain and jaundice to weight loss, making diagnosis challenging. The clinical course can be indolent or aggressive, depending on the tumor growth [7].

Diagnosis involves clinical, radiological, and histopathological findings, with imaging modalities such as magnetic resonance imaging (MRI), computed tomography (CT), and endoscopic retrograde cholangiopancreatography (ERCP) playing crucial roles. Endoscopic ultrasound (EUS) and intraductal ultrasound (IDUS) aid in lesion evaluation, while definitive diagnosis requires histopathological examination from biopsy or surgical resection [8].

The management of IPNBs requires a multidisciplinary approach, including surgical resection, adjuvant therapies, and long-term surveillance tailored to tumor characteristics and patient factors. Surgical resection is the cornerstone for localized IPNBs, with adjuvant therapies for high-risk or unresectable disease.

Prognosis depends on tumor stage, histological subtype, and surgical resection extent. IPNBs generally have a favorable prognosis compared to CCAs, especially when localized and surgically removed. However, the risk of recurrence and progression necessitates long-term surveillance and comprehensive management. Recurrence rates range from 13% to 62%, highlighting the need for early recognition and treatment strategies against CCA [3].

## 2. Case Report 1

**Figure 1:**
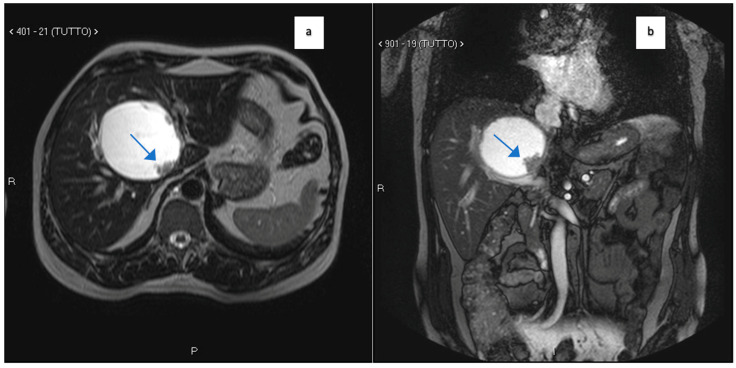
(**a**,**b**): MRI T2 axial (**a**) and coronal (**b**) images showing a large cystic mass involving the IV hepatic segment. An irregular-shaped mass is present on the posterior boundary of the cyst (arrows). The portal vein appears dislocated downward by the cyst.

**Figure 2:**
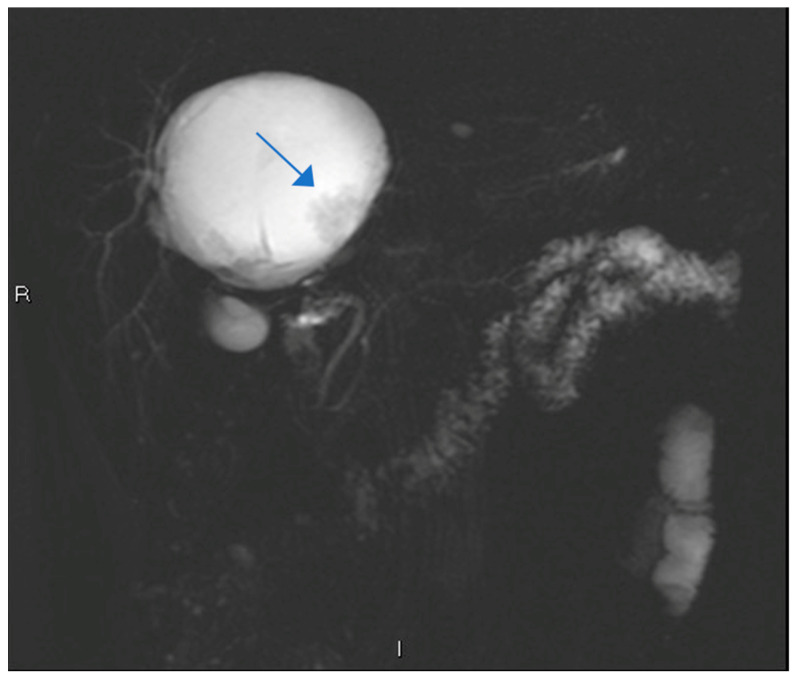
MR cholangiography showing a large, well-defined mass in close proximity to the biliary tree. The mass appears to cause displacement of the biliary ducts, suggesting a potential compressive effect rather than direct invasion.

A 60-year-old Caucasian male was referred to our Hepatobiliary Unit in 2013 following the incidental discovery of an 8 × 8 × 9 cm round-shaped hepatic cyst located in segments S-IV, S-VII, and S-VIII. MRI revealed the cyst’s characteristics, including thin regular walls, internal *septa*, papillary projections, and enhancing solid components (Figure 1). Magnetic resonance cholangiopancreatography (MRCP) revealed a mass that displaced the biliary ducts, suggesting a potential compressive effect rather than direct invasion (Figure 2). This spatial relationship indicated that, while the mass was located near the biliary tree, further histopathological correlation was required to determine the nature of their interaction. The observed displacement pointed to extrinsic compression of the biliary tree, consistent with the effects seen in adjacent vascular structures, such as the portal vein in Figure 1.

The patient’s past medical history included HBV infection (with HBsAg negativity at consultation), benign prostatic hyperplasia (BPH), significant coronary artery stenosis, nodular thyroidopathy, and an allergy to Piperacillin-Tazobactam. Blood tests revealed normal bilirubin levels but elevated levels of AST (79 mg/dL, normal range 6–32), ALT (245 mg/dL, normal range 15–56), ALP (388 UI/L, normal range 40–129), and γ-GT (606 UI/L, normal range 5–85). While hepatobiliary and pancreatic onco-markers were negative, a mild positivity for Echinococcus Granulosus (IgG: 0.9, mild positivity range 0.9–1.1) was observed, prompting a provisional diagnosis of a hydatid cyst. This provisional diagnosis, due to the suspicion of an echinococcal infection, led to the decision for the patient to undergo surgery for cyst resection and cholecystectomy. A subsequent ERCP, requested despite the already-performed MRCP, was conducted to further clarify the optimal surgical strategy and revealed an anatomical variant of the biliary tree with migration of the posterior right bile duct to the left hepatic duct (segments VI and VII).

Intraoperatively, a large partially exophytic floating soft mass was found and successfully removed with good margins. Postoperatively, the patient experienced an early bile leak from the segment-IV duct, which was managed endoscopically with multiple stenting. The patient was discharged on the 11th postoperative day, and the stents were later removed at the 1-month follow-up.

**Figure 3:**
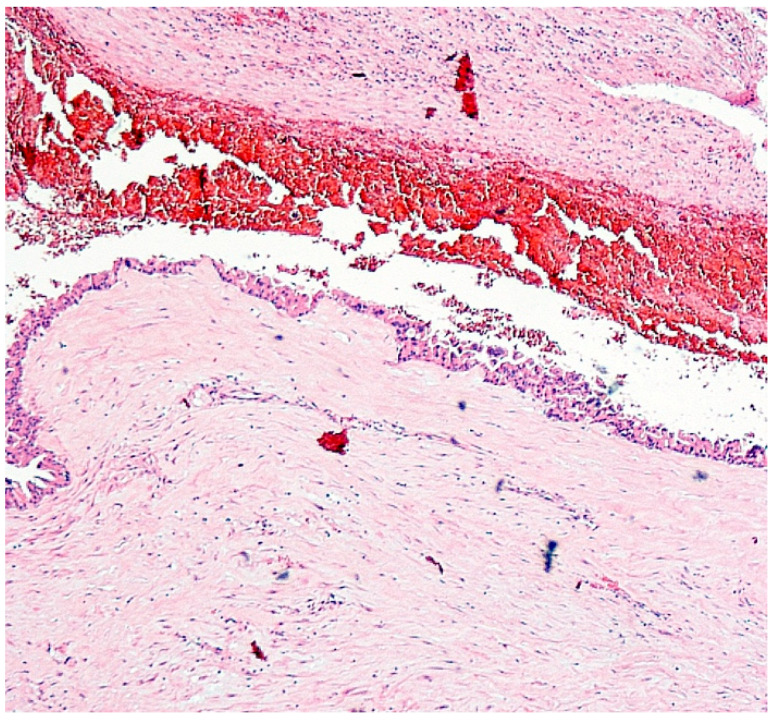
EE 100× cystic adenoma. Lining epithelium was composed of cuboidal and columnar cells, many of which had an abundant eosinophilic cytoplasm producing an oncocytic appearance.

**Figure 4:**
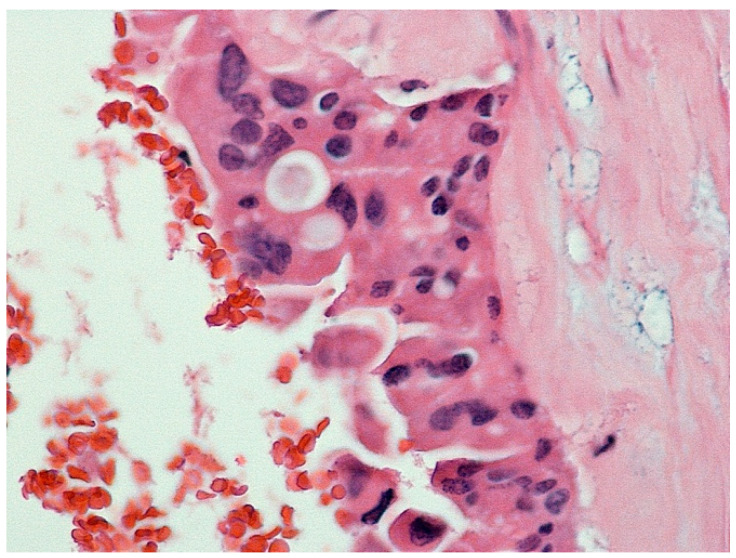
EE 400× epithelial cells showed varying degrees of dysplasia, in some places high-grade.

Histopathological examination revealed challenging findings due to the fibrous wall with incomplete lining epithelium, interrupted by areas of ulceration, hemorrhage, and granulation tissue formation. Notably, the lining epithelium displayed typical characteristics of IPNB, exhibiting an oncocytic appearance and varying degrees of dysplasia (Figure 3). Additionally, a larger polypoid projection into the cystic lumen was observed in one area, along with epithelial cells showing varying degrees of dysplasia (Figure 4). Mucus was indeed found within the cyst, which is consistent with the characteristics of IPNB.

Immunohistochemical analysis of the epithelial cells revealed strong diffuse immunoreactivity for Mucin-1 (MUC-1) and mitochondrial antigen, along with moderately extensive positivity for MUC-2 and cytokeratin 7 (CK7). Furthermore, staining was focally positive for hepatocyte-specific antigen and weakly positive for CK20, while being negative for CDX2. The final immunohistochemical profile exhibited pan-CK+, CK19+, CK7+, and EMA+ cells, with 70% HSA+ cells and a Ki67 proliferation index of 30%.

A conclusive diagnosis of intraductal papillary neoplasms of the bile duct with small foci of adenocarcinoma with negative oncologic margins was established. After multidisciplinary discussion, no adjuvant chemotherapy was indicated, and the patient initiated routine oncological follow-up. This follow-up included regular MRCP at six months, one year, and then annually for five years to monitor for intraductal lesions. Subsequent follow-up was continued with ultrasound examinations. Endoscopic procedures were not deemed necessary due to the negative findings on radiological imaging. The patient was discharged after five years.

**Figure 5:**
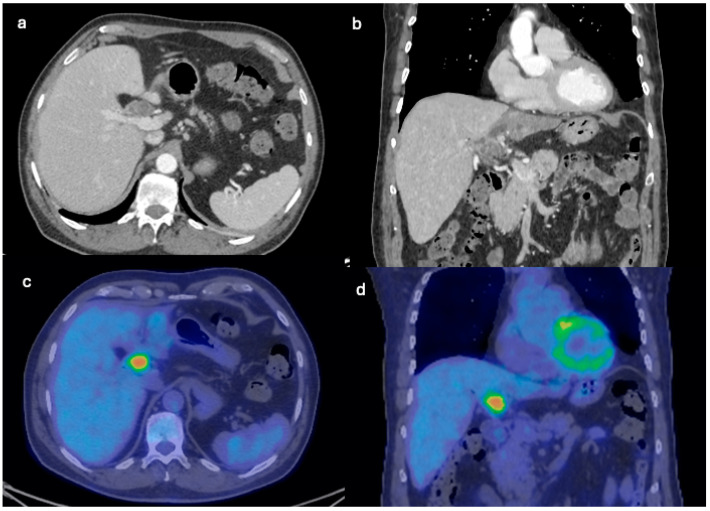
CT axial and coronal planes on parenchymal window showing a multicystic lobulated mass involving the hepatic hilum. After injection of iodinated contrast, the mass appears hypo-vascular, with enhancing margins and internal septations (**a**,**b**). PET axial and coronal images show increased uptake of ^18^[F]-fluorodeoxyglucose in the multicystic lobulated mass involving the hepatic hilum suspicious for recurrences of IPBN (**c**,**d**).

**Figure 6:**
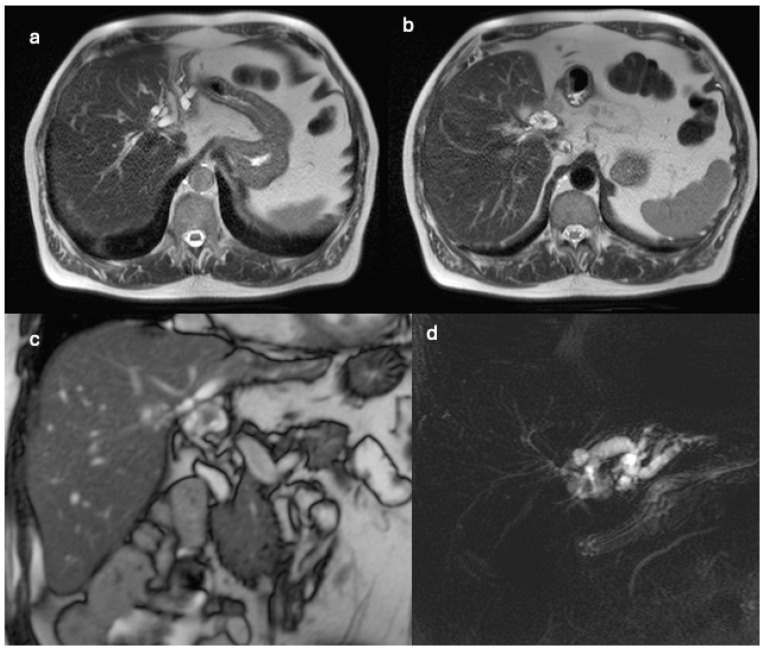
MRI T2 axial (**a**,**b**), T2 coronal (**c**), and MR cholangiography (**d**) images showing a multicystic lobulated mass with internal septations involving the hepatic hilum and causing dilation of the intrahepatic biliary ducts.

Three years after the last follow-up, in June 2021, the patient presented with fever and malaise due to cholangitis. Elevated levels of Ca19.9 (463 UI/mL, normal range 0–35) were observed, with normal α-FP and CEA. Subsequent imaging studies, including EUS and positron emission tomography (PET)-CT scan (Figure 5), revealed a metabolically active lesion involving the hepatic hilum. Given the specific location and metabolic activity, a de novo-type IIIb Klatskin tumor was initially suspected. However, further evaluation with MRI provided additional insights: the MRI T2 axial and coronal images, along with MR cholangiography (Figure 6), demonstrated a multicystic lobulated mass with internal septations involving the hepatic hilum, causing dilation of the intrahepatic biliary ducts. These imaging findings were crucial in guiding the next steps in diagnosis and management. Subsequent biopsy via endoscopic retrograde cholangiopancreatography (ERCP) confirmed the diagnosis of recurrent papillary mucinous neoplasia.

Multidisciplinary discussion recommended surgical intervention, leading to a left hepatectomy with lymphadenectomy. During the surgical procedure, an extensive lymph node dissection was performed, including the removal of lymph nodes from the hepatoduodenal ligament, the celiac axis, and the para-aortic region. Intraoperative ultrasound revealed no additional hepatic lesion, and no distant peritoneal disease was detected during abdominal exploration. The postoperative course was complicated by a bile collection diagnosed with CT scan on POD 4 and treated by percutaneous drain positioning, which was removed on POD 15 when the patient was discharged in good clinical condition.

Histopathological examination of the resected specimen confirmed a rT2aN0 type-1 oncocytic intraductal papillary neoplasia of the bile ducts with mucinous aspects and high-grade dysplasia and foci of invasive adenocarcinoma invading the peri-periductal adipose tissue. The neoplasia showed dishomogeneous positivity for MUC6 and HSA, with a Ki67 index of 30%.

Considering the pathological staging, adjuvant chemotherapy was not recommended, and the patient commenced routine follow-up. At 33 months post-surgery, the patient remained alive and in good clinical condition.

## 3. Case Report 2

A 28-year-old Caucasian female with a medical history of cognitive impairment due to hypoxic-ischemic encephalopathy was admitted to our hospital following a prior diagnosis of pulmonary embolism and a liver space-occupying lesion, identified on CT conducted elsewhere. Abdominal US imaging conducted years earlier had incidentally revealed a simple cyst-like lesion in the liver’s segment S-IV, characterized by regular walls.

Upon admission, she presented with dyspnea, vomiting, jaundice, and fever. Physical examination revealed a soft abdomen, without tenderness, rebound tenderness, or a palpable abdominal mass in the upper quadrants. Laboratory findings revealed the following abnormalities: hemoglobin was 9.3 g/dL (normal range: 12–16 g/dL); white blood cell count was 14,880/mm^3^ (normal range: 4300–10,800/mm^3^); INR was 1.41 (normal range: 0.8–1.2); and D-dimer level was 21,238 ng/mL (normal range: 0–500 ng/mL). Liver function tests showed hypoalbuminemia, 2.18 g/dL (normal range: 3.5–5.1 g/dL); direct bilirubin, 1.58 mg/dL (normal range: <0.5 mg/dL); total bilirubin, 1.83 mg/dL (normal range: <1.2 mg/dL); normal transaminases: AST was 34 U/L (normal range: 5–34 U/L); ALT was 19 U/L (normal range: 0–55 U/L); elevated ALP, 508 IU/L (normal range: 40–150 U/L); and elevated γ-GT, 206 IU/L (normal range: 8–33 U/L). Renal function was normal, with BUN: 30 mg/dL (normal range: 15–40 mg/dL) and creatinine 0.58 mg/dL (normal range: 0.55–1.02 mg/dL). Serum levels of tumor markers were as follows: CEA: 317.29 ng/mL (normal range: <5 ng/mL); α-FP: 0.92 IU/mL (normal range: 0.00–6.72 IU/mL); and Ca 19.9: 20,000 U/mL (normal range: 0–35 U/mL).

Chest CT confirmed bilateral diffuse pulmonary embolism with pleural and pericardial effusions, while abdominal CT revealed a significantly enlarged liver with an irregular mass displaying inhomogeneous contrast enhancement, measuring approximately 10 cm in its greatest dimensions, previously misdiagnosed as a hemangioma. The intrahepatic bile ducts were dilated due to the volume effect of the lesion, and the intrapancreatic portion of the common bile duct was also dilated (10 mm). Additionally, right ilio-femoral venous thrombosis was confirmed, leading to the initiation of intravenous administration of antibiotics and subcutaneous administration of low-molecular-weight heparin.

**Figure 7:**
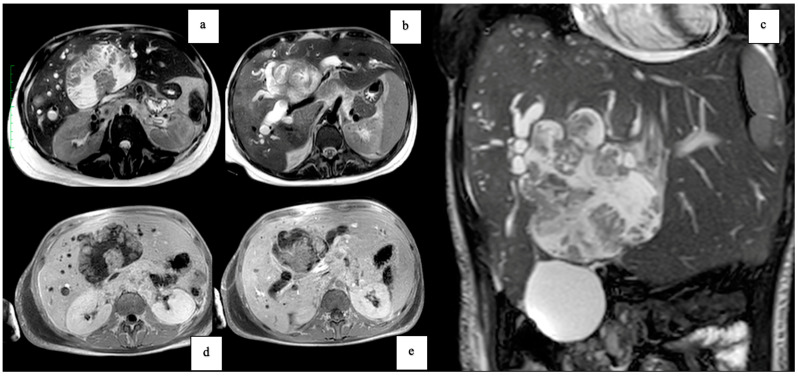
(**a**–**c**): MR images with T2 sequence in axial and coronal planes revealing a 10 cm diameter solid cystic lesion with vegetations along its walls. The images illustrate marked dilation of the intrahepatic bile ducts associated with the lesion. Additionally, diffuse signal alteration and centimetric nodules within the surrounding hepatic parenchyma suggest widespread disease localization; (**d**,**e**): axial T1-weighted images, post-intravenous administration of paramagnetic contrast agent, showcasing enhancement of the solid components of the lesion, internal vegetations, and the surrounding parenchyma infiltrated by the disease.

MRI unveiled a large lesion in the left hepatic lobe (S-IV), demonstrating hyperintensity in T2-weighted images (WI), hypointensity in T1WI, and restricted diffusion in DWI. The lesion contained hypointense fluid-containing components, with hypointense T2WI components along the wall exhibiting a protuberant appearance and pronounced contrast enhancement (Figure 7). Moreover, MRI revealed T2-weighted hyperintensity, diffusion restriction on DWI, and enhancement following intravenous contrast administration in the superior region of the liver. Furthermore, two small nodules were identified in S-IV and S-VI, indicating intrahepatic metastases. Compression of the hepatic artery and the portal venous system was observed, with thrombosis of the portal vein not definitively ruled out. Lymphadenopathy of the para-aortic and caval lymph nodes, as well as of lymph nodes around the coeliac axis and porta hepatis, was also noted, suggesting lymph node metastases. Finally, a thrombus in the inferior vena cava (IVC) was detected, leading to the placement of an IVC filter for the patient’s management. MRCP illustrated biliary tree dilatation in the left lobe.

**Figure 8:**
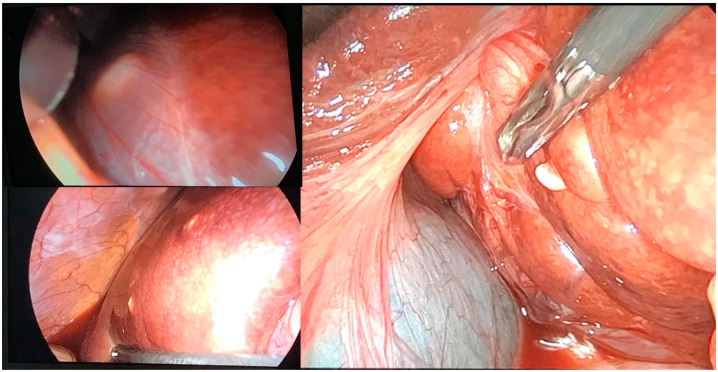
Laparoscopic view of the lesion.

A laparoscopic liver biopsy was performed (Figure 8), specifically targeting the small tumorous lesions in S-IV and S-VII. This approach was chosen to minimize the risk of bleeding and peritoneal dissemination associated with targeting the cystic wall of the main tumor.

Histological examination of the biopsy specimens revealed the presence of adenocarcinoma cells with papillary, clear cell, and mucinous appearance, sometimes exhibiting gland ectasis within pools of mucin and cystic or pseudocystic aspects.

**Figure 9:**
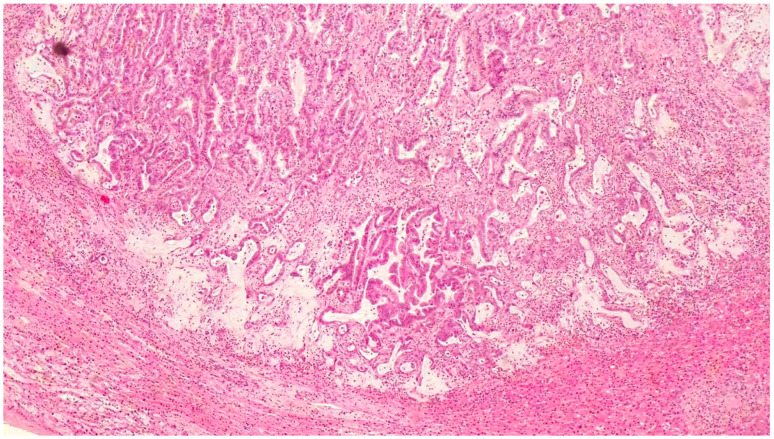
Histological characteristics of the tumor. Notice the papillary features with pools of mucin and a cystic/pseudocystic appearance. The neoplasm exhibits strong acute inflammation and areas of necrosis. Based on its immunohistochemical profile, the diagnosis of IPNB was confirmed.

Immunohistochemical analysis showed that the tumor cells expressed several markers including CK7, CK19, MUC5AC, MUC1, CEA, EMA, MOC31, and CAM 5.2. Additionally, some cells expressed hepatocyte-specific antigen (HSA) and caudal-type homeodomain transcription factor 2 (CDX2). However, other markers, such as CK20, estrogen receptor (ER), GATA3, Wilms tumor gene-1 (WT1), PAX8, p53, VIM, and CD10, yielded negative results. Notably, no structures indicating ovarian-like stroma were found in the biopsy tissue specimens. Furthermore, chronic cholecystitis and adenocarcinoma features of the cystic duct lymph node were detected. Histological examination of the hepatic artery lymph node revealed adenocarcinomatous metastases. The pathological characteristics of the tumor, including the papillary structure, mucin production, and immunohistochemical profile, were consistent with IPNB (Figure 9).

During a transesophageal echocardiography, a thrombus in the right atrium was detected. However, due to the terminal stage of the patient’s condition, surgical intervention was deemed inappropriate. Unfortunately, the patient eventually died from hepatic failure.

A comprehensive summary of the key histopathological findings and clinical characteristics from both cases is presented in Table 1.

## 4. Discussion

IPNBs represent a rare subtype of premalignant neoplasms characterized by papillary growth within the bile ducts. Despite historical comparisons with intraductal papillary neoplasms of the pancreas (IPMN), IPNBs exhibit unique clinicopathological features, establishing them as a distinct entity. Major risk factors for IPNBs vary geographically, with hepatolithiasis, liver fluke infections, and occupational exposure to printing solvents being prevalent in East Asian countries, while congenital biliary tract diseases and primary sclerosing cholangitis (PSC) are more common in Western countries and are associated with more invasive disease [9,10].

The pathogenesis of IPNBs involves a complex interplay of genetic, environmental, and inflammatory factors, leading to chronic biliary inflammation, bile stasis, and genetic alterations in oncogenes and tumor suppressor genes. In 2019, the World Health Organization (WHO) classified IPNB into four subtypes based on epithelial cell lineages, including intestinal (iIPNB), gastric (gIPNB), pancreato-biliary (pbIPNB), and oncocytic (oIPNB) subtypes, based on epithelial cell lineage and mucin expression profiles [5,11]. Subtyping relies on the detection of intracellular mucin expression through immunohistochemistry, including markers such as CK20, MUC5AC, and MUC2. iIPNB and pbIPNB are relatively more prevalent compared to gIPNB and oIPNB, with geographical variations in incidence. Intrahepatic IPNBs predominantly manifest as the intestinal subtype, while extrahepatic IPNBs commonly present as intestinal or pancreato-biliary subtypes [1].

Neoplastic epithelial cells in IPNBs exhibit varying degrees of atypia, from low-grade to high-grade dysplasia, culminating in malignant transformation and invasive carcinoma. Approximately between 10% and 40% of IPNBs manifest as low-grade lesions, while high-grade IPNBs are associated with invasive adenocarcinoma in up to 74% of cases [5]. The Japan–Korea Pathologist Group proposed a subclassification of IPNBs into two types: Type 1, a “non-invasive phenotype” with regular structures and a favorable prognosis, and Type 2, an “invasive phenotype” characterized by irregular structures and a higher propensity for invasive carcinoma [12].

IPNBs are recognized as precursors to cholangiocarcinoma (CCA), along with biliary intraepithelial neoplasms (BilIN) and hepato-biliary mucinous cystic neoplasms (MCN) [13,14,15]. The prevalence of IPNB among bile duct neoplasms ranges from 4% to 15%, and they can occur in both intrahepatic and extrahepatic bile ducts [16,17,18].

Clinically, IPNBs typically present in patients aged between 50 and 70 years, with a slight male predominance. Common symptoms include acute cholangitis, jaundice, and abdominal pain, although up to 30% of patients may be asymptomatic at diagnosis [19].

Imaging studies such as ultrasound (US), CT, and MRI are crucial for diagnosing IPNB, revealing characteristic features such as intraductal masses and bile duct dilatation [20,21,22,23,24,25,26].

Several articles describe various characteristics and sizes of the cystic lesions associated with intraductal papillary neoplasms of the bile duct (IPNB), emphasizing their diversity and complexity. For instance, Zulfiqar et al. [27] reported on the imaging features of premalignant biliary lesions, including large cystic lesions, which can vary significantly in size and presentation. Aparicio Serrano et al. [28] highlighted the malignancy potential of these cystic lesions, underscoring their clinical importance and the need for accurate diagnosis. Aliyev et al. [29] described a case of a huge IPNB treated by right trisectionectomy after right portal vein embolization, demonstrating the surgical challenges posed by such large cystic masses. Watanabe et al. [30] documented an oncocytic variant of IPNB that formed a giant hepatic cyst, further illustrating the wide range of cystic presentations in IPNB patients.

Given the high risk of malignant transformation, early surgical resection is the mainstay of treatment for IPNB, aiming for complete (R0) resection. The prognosis for IPNB patients is generally more favorable than for those with conventional CCA, with recurrence-free survival rates of 93.8% at 1 year and 70% at 5 years [7]. However, factors such as older age, lymph node metastasis, and high CA19-9 levels are associated with worse outcomes [5]. In addition to CA19.9, other markers, such as carcinoembryonic antigen (CEA), alpha-fetoprotein (AFP), and various mucins (e.g., MUC1 and MUC5AC), have been investigated. Combinations of these markers may improve diagnostic accuracy. For example, the combined use of CA19.9 and CEA has shown higher specificity in some studies, while MUC5AC expression is particularly useful in distinguishing IPNB from other biliary lesions [31].

Recent advances in the understanding of IPNB pathogenesis have paved the way for innovative approaches in diagnosis and treatment. One promising area of research involves the use of next-generation sequencing (NGS) to identify specific genetic mutations associated with IPNB, such as mutations in the KRAS, GNAS, and CTNNB1 genes. These genetic markers can not only help in early detection but also guide targeted therapies aimed at specific molecular pathways involved in IPNB progression [32].

Additionally, the development of minimally invasive techniques, such as advanced endoscopic ultrasound (EUS) and peroral cholangioscopy (POCS), has improved the diagnostic accuracy and therapeutic management of IPNB. These techniques allow for precise localization and characterization of lesions, facilitating targeted biopsies and localized treatments such as radiofrequency ablation (RFA) or photodynamic therapy (PDT), which can be particularly beneficial for patients who are not candidates for surgery [33].

Immunotherapy is another emerging field in the treatment of IPNB. Checkpoint inhibitors, which have shown success in various malignancies, are being explored for their potential efficacy in treating high-grade dysplastic IPNBs and invasive CCAs arising from IPNBs. Early clinical trials have demonstrated promising results, indicating that immunotherapy could become an integral part of the therapeutic arsenal against IPNB [34].

Furthermore, ongoing research into the microbiome’s role, especially in CCA, suggests that modulating the gut–liver axis might offer new preventive and therapeutic strategies for IPNB. Probiotics, antibiotics, and other microbiome-targeting therapies could potentially reduce chronic inflammation and lower the risk of malignant transformation in IPNB patients [35,36].

## 5. Conclusions

IPNBs present a unique challenge in hepatobiliary oncology due to their premalignant nature and potential for aggressive behavior. Optimizing outcomes and enhancing survival in IPNB patients necessitates comprehensive, multidisciplinary approaches. Further research is warranted to elucidate the underlying mechanisms of IPNB pathogenesis and develop novel therapeutic strategies. International collaborations and registries are crucial for advancing our understanding of IPNB and refining treatment protocols to enhance patient outcomes. R0 surgical resection typically yields favorable oncological outcomes with minimal risk of cancer recurrence. The surgical intervention for localized, resectable recurrences is both safe and feasible in highly specialized centers.

Our two case reports illustrate the diverse clinical presentations and outcomes of IPNB. The first case highlights the diagnostic challenges and the importance of long-term surveillance, while the second case underscores the aggressive nature of some IPNB variants and the need for early detection and intervention. Both cases emphasize the critical role of multidisciplinary collaboration in managing IPNB and the importance of continued research to better understand and treat this complex disease.

By integrating novel diagnostic and therapeutic approaches, such as genetic profiling, minimally invasive techniques, immunotherapy, and microbiome modulation, we can improve patient outcomes and advance the management of IPNB. These innovative strategies not only enhance our understanding of IPNB but also offer new hope for more effective and personalized treatment options.

## Figures and Tables

**Table 1:** Summary of case reports.

Feature	Case 1	Case 2
Patient Demographics	60-year-old Caucasian male	28-year-old Caucasian female
Clinical Presentation	Incidental discovery of an 8 × 8 × 9 cm hepatic cyst	Presented with dyspnea, vomiting, jaundice, fever
Initial Diagnosis	Suspected hydatid cyst	Simple cyst, later adenocarcinoma with metastases
Diagnostic Methods	MRI, MRCP, ERCP, histopathological examination	CT, MRI, laparoscopic liver biopsy, histopathological examination
Tumor Characteristics	Large cystic mass, thin walls, internal septa, papillary projections, enhancing solid components	Large lesion in left hepatic lobe, hyperintensity in T2-weighted images, restricted diffusion, contrast enhancement
Treatment	Cyst resection, cholecystectomy	Thrombectomy, chemotherapy, supportive care
Surgical Findings	Partially exophytic floating soft mass	Extensive intrahepatic metastases, vascular involvement
Histopathological Findings	IPNB with foci of adenocarcinoma, oncocytic appearance, varying degrees of dysplasia, mucus within cyst	Adenocarcinoma with papillary clear cell and mucinous appearance, gland ectasis, cystic or pseudocystic aspects
Follow-up and Outcome	Initial recurrence-free survival for 8 years, recurrence treated with left hepatectomy, patient alive at 33 months post-second surgery	Disease progression despite aggressive management, patient died from hepatic failure

## Data Availability

The original contributions presented in the study are included in the article; further inquiries can be directed to the corresponding author.
